# South Asian immigrants’ and their family carers’ beliefs, practices and experiences of childhood long‐term conditions: An integrative review

**DOI:** 10.1111/jan.15217

**Published:** 2022-03-14

**Authors:** Indu Sudarsan, Karen Hoare, Nicolette Sheridan, Jennifer Roberts

**Affiliations:** ^1^ Massey University Wellington New Zealand; ^2^ Elderslea Rest Home Wellington New Zealand; ^3^ School of Nursing Massey University, Albany Auckland New Zealand; ^4^ Greenstone Family Clinic, Manurewa Auckland New Zealand; ^5^ College of Healthcare Sciences James Cook University Townsville Australia

## Abstract

**Aim:**

The aim was to examine South Asian immigrants' beliefs, practices and experiences of childhood long‐term conditions.

**Design:**

This was an integrative review.

**Data sources:**

MEDLINE, PsycINFO, PubMed, Scopus and Web of Science were searched for primary peer‐reviewed articles published in English between January 2011 and April 2021.

**Review methods:**

Articles were screened based on PRISMA guidelines. The quality of the studies was evaluated using the Critical Appraisal Skills Programme Checklist for qualitative studies and the Joanna Brigg's Institute Critical Appraisal Checklist for quantitative studies.

**Results:**

Fourteen studies were included in the review. South Asian immigrant children and their family carers experienced cultural clashes as they attempted to incorporate their cultural beliefs about long‐term conditions into a more westernized biomedical approach. Families were overburdened by caregiving and struggled to find additional support for their children. The main findings were categorized into three themes: (1) cultural beliefs; (2) religious, spiritual and complementary and alternative medicine practices and (3) care and support of the child.

**Conclusion:**

Health‐care providers should use a combination of culturally safe management strategies and a nuanced approach to educational initiatives on the biomedical aspects of various long‐term conditions to effectively engage South Asian immigrant families with health services.

**Impact:**

The growth of South Asians worldwide along with the increased burden of long‐term conditions among South Asian immigrant children has implications for health service delivery. However, no reviews to date have explored South Asian immigrants' experience of childhood long‐term conditions. Incorporating South Asian immigrants' beliefs and practices into the plan of care promotes collaborative decision‐making that can lead to better treatment adherence, improved health outcomes and higher patient and family satisfaction. The findings encourage clinicians, researchers and policymakers to develop culturally safe child/family‐centred interventions to address the specific needs of South Asian immigrant children with long‐term conditions.

## 1 INTRODUCTION

South Asian (SA) countries continue to be the main sources of international migration (Batra et al., 2019). Geographically, South Asia comprises countries such as Pakistan, India, Nepal, Bhutan, Bangladesh and Sri Lanka (Mann, 2014). However, countries differ in their definition of the SA group. For instance, in New Zealand, the term SA also includes people of SA descent who are from Malaysia, Fiji and other countries (Didham, 2010). In the United Kingdom (UK), the SA group refers mainly to the population from the Indian subcontinent (Liu et al., 2016). Despite geographic, religious and linguistic differences, SAs share many unique cultural norms and social values that may influence their health‐care beliefs and practices (Ramaswamy et al., 2019). The growth of SAs worldwide along with the increased burden of long‐term conditions (LTC) among children has implications for health service delivery (Liu et al., 2016). In addition to the difficulties encountered by any parent of a child with a LTC, the context of being an immigrant makes some experiences, especially daunting (Englund & Rydström, 2012).

## 2 BACKGROUND

SA immigrant children are particularly vulnerable in terms of morbidity and mortality from LTC (Lakhanpaul et al., 2020; Zechella & Raval, 2016). For example, SA children in the UK are more likely to present with uncontrolled asthma symptoms and become hospitalized with acute asthma exacerbations compared with their White British counterparts (Lakhanpaul et al., 2020). These hospitalizations are often identified as potentially preventable. Similar ethnic disparities in asthma outcomes have also been noted in the USA, New Zealand and Canada (Benchimol et al., 2015; Lakhanpaul et al., 2020; Mehrotra et al., 2014; Mehta, 2012).

Health disparities among SA immigrants may be the result of beliefs and practices that contrast with that of health‐care professionals' (HCP) advice who are obliged to follow evidence‐based (EB) guidelines where available (Lakhanpaul et al., 2019). The philosophy of child‐ and family‐centred care (CFCC), which has been widely practiced in paediatrics for decades, is central to reducing health disparities (Ford et al., 2018; Gerlach & Varcoe, 2020; Watt et al., 2011). CFCC involves HCPs listening to and respecting families' and children’s perspectives, ensuring that their priorities, knowledge and beliefs are appropriately integrated into all aspects of care planning and delivery, as well as providing accessible and timely information that supports their participation in decision‐making at a level suitable for their maturity and understanding (Gerlach & Varcoe, 2020). However, a growing body of literature indicates an inconsistency in the implementation of CFCC in practice, particularly among immigrants. This could be due to several factors, including a lack of culturally congruent care, insufficient organizational support, budget constraints and cuts to human resources and services. The challenge for health‐care services is to maintain a balance between the child's best interests, respect for the family and community as a unit and professional expertise to provide optimal care for the child (Watt et al., 2011).

With the large‐scale migration of SAs worldwide, more HCP will encounter this group of people. No reviews to date have explored SA immigrants' experience of childhood LTC. However, a few reviews that examined the experiences of childhood LTC among SAs included studies on SAs in both minority and majority settings. Although there is some degree of generalizability of these findings to SA immigrants, it may not be an accurate representation of their illness experience in their host country. SAs may experience ongoing social and cultural transitions because of migration, which may impact their health behaviour and utilization of healthcare (Ahmed et al., 2018). Therefore, exploring SAs' cultural needs and incorporating them in the host country’s model of care becomes crucial to the optimal management of childhood LTC. To the best of our knowledge, the current review is the first of its kind to explore SA children's and their family carers' experience of LTC in their host country.

Simultaneously, listening to the voices of SA immigrant children with LTC is as important as that of their family carers to effective management (Lakhanpaul et al., 2019). However, studies on childhood LTC among SA immigrants predominantly focus on the voices of family caregivers and HCP. Only a few studies seek to give voice to SA immigrant children. This is despite Article 12 of the United Nations Convention on the Rights of the Child (UNCRC) which articulates the significance of giving voice to children in all matters affecting them (Lakhanpaul et al., 2019). The current review addresses this gap by examining studies with first‐ and second‐generation SA immigrant children and young people, as well as studies involving first‐generation SA family caregivers.

## 3 CONCEPTUAL FRAMEWORK

The tenets of *Social Constructionism* served as the conceptual framework to conduct this review. First introduced by Berger and Luckmann, social constructionism is based on the principle that the meanings of social reality are not discovered but constructed by people as they interact in a given context. This concept is widely used to explain the concept of illness (Berger & Luckmann, 1991; Burr, 2003; Conrad & Barker, 2010). Burr (2003) considers illness as a socially constructed phenomenon rather than a fixed physiological entity. Perceptions of illness vary with the values, beliefs and norms of the group of people being studied. Social constructs related to illness differ not only with groups of people but also with place and time (Berger & Luckmann, 1991; Burr, 2015). For example, people's constructs about an illness may vary as they move settings such as when they migrate. There may also be a change in these constructs as they experience illness over time. For instance, following diagnosis, people may regularly seek and process new information about the condition which may result in a change in attitude towards the illness and resultant behaviour (Burr, 2015; Gergen, 2015; Gupta, 2010). The current integrative review posits that SA immigrant children with LTC and their family caregivers construct their own interpretations of reality and shared meanings about various LTC. These constructs exist in a specific social, cultural and historical context and may change over time (Burr, 2015; Gergen, 2015).

## 4 THE REVIEW

### 4.1 Aim

The aim was to synthesize primary research on SA immigrant children's and their family carers' beliefs, practices and experiences of childhood LTC.

### 4.2 Design

The modified integrative review framework developed by Whittemore and Knafl (2005) guided this literature review. The main feature that distinguishes an integrative review from other types of literature reviews is its scope; the flexibility in including diverse methodologies (both quantitative and qualitative), empirical and theoretical literature. High‐quality integrative reviews can guide the development of evidence‐based policy and practice initiatives and make recommendations for future research (Knafl & Whittemore, 2017). Integrative reviews play a key role in transcultural nursing as they allow exploration of complex inherent concepts related to the health‐related beliefs and practices of people from diverse backgrounds (Whittemore & Knafl, 2005). However, the key challenge in conducting an integrative review is ensuring rigour as it combines a large volume of data from primary research that employs various methodologies. To address this issue, Whittemore and Knafl (2005) and Knafl and Whittemore (2017) developed systematic methodological strategies specifically for each stage of the review process, thus making it popular as a standard framework for undertaking integrative reviews.

### 4.3 Search methods

Five electronic databases were searched: CINAHL, MEDLINE, PsycINFO, PubMed, Scopus and Web of Science. The following keywords and phrases were used: *asthma, wheeze, respiratory, eczema, cancer, diabetes, autism, developmental, congenital, illness, sickness, health, chronic, long term, South Asia, India, Pakistan, Bangladesh, Punjab, Gujarat, child, paediatric, adolescent, teenage, young adult, migrant, immigrant, care giver, carer, caregiver, parent and family*. Modifications were made to the search strategy to fit each database. Boolean operators such as ‘AND’ and ‘OR’ combined the keywords. In some databases, truncations such as asterisks (*) were used with the root forms of the keywords to retrieve all the related variant terms and quotation marks (““) to indicate phrases (See Table S1). The database search was limited to the years 2011–2021 due to two reasons; firstly, to review the most up‐to‐date studies and secondly, due to the last decade seeing the largest migration of SAs (International Organisation for Migration, 2019). The online database search was supplemented by ancestry searching, citation searching and manual searching of selected journals. Table 1 lists the inclusion and exclusion criteria applied to the literature search.

**TABLE 1:** Inclusion and exclusion criteria

Inclusion criteria	Exclusion criteria
Participants: SA immigrant children (0–18 years) and young people (10–24 years) (World Health Organization [WHO], 2014) with LTC and/their family carers. Includes one of the following: attitudes, beliefs, practices, experiences or perceptions of children and/ family caregivers. Primary, peer‐reviewed studies in English and published between January 2011 and April 2021.	Intervention studies. Grey literature (policy, proceedings, etc).

### 4.4 Search outcome

**FIGURE 1:**
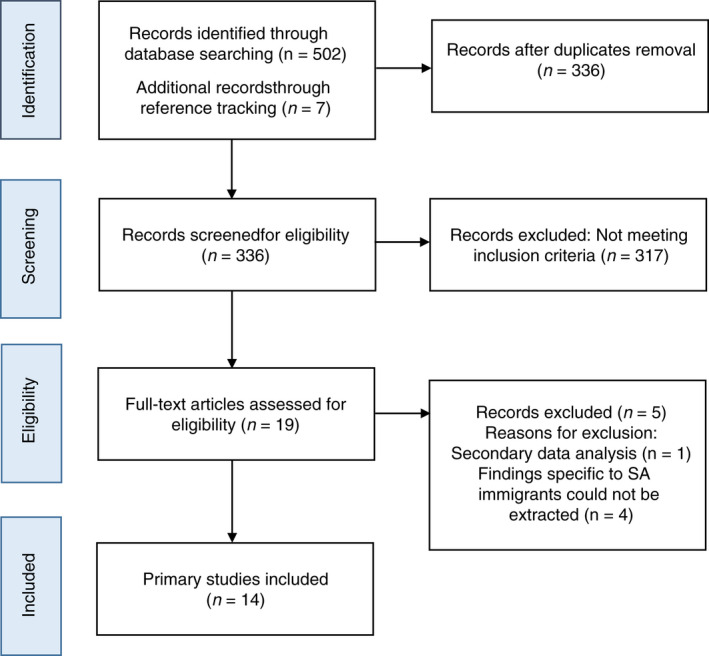
PRISMA flow diagram of the literature search process (Moher et al., 2009)

Preferred Reporting Items for Systematic reviews and Meta‐Analyses (PRISMA) guidelines were used for the article selection and screening procedure (Moher et al., 2009) (See Figure 1). The first two authors, Indu Sudarsan (IS) and Karen Hoare (KH), independently examined the titles and abstracts from the initial search. Those articles that met the eligibility criteria were chosen for full‐text review (See Table S2). IS and KH separately reviewed the full‐text articles and then jointly if there was any doubt or disagreement.

### 4.5 Quality appraisal

The quality of the studies was evaluated using the Critical Appraisal Skills Programme Checklist (2018) for qualitative studies and the Joanna Brigg's Institute (2020) Critical Appraisal Checklist for quantitative studies (See Tables S3 and S4). No studies were excluded following the quality appraisal.

### 4.6 Data abstraction and synthesis

A data display table was used to present the data extracted from each study which included information on the author, year of publication, country, study objectives, sample characteristics, chronic condition, study design, research methods and key findings. Data analysis involved identifying the constructs of beliefs, practices and experiences from each study. A constant comparison approach was employed to facilitate the identification of patterns, variations, themes and relationships when analysing the data. The main findings were categorized into three themes: (1) cultural beliefs; (2) religious, spiritual and complementary and alternative medicine practices and (3) care and support of the child.

## 5 RESULTS

### 5.1 Characteristics of the studies

Table 2 shows the characteristics of the 14 studies included in the final review. Most studies were qualitative (*n* = 13) and used a wide range of methodologies such as Interpretative Phenomenological Analysis (IPA), Grounded Theory (GT), case report, descriptive design, constructivist interpretative paradigm and ethnography. The only quantitative study included in the review used an online survey. Most studies were conducted in the UK (*n* = 8) with the rest conducted in the United States (*n* = 4), Canada (*n* = 1) and Ireland (*n* = 1). The LTC included: Asthma (*n* = 3), autism (*n* = 3), cancer (*n* = 1), beta‐thalassemia major (*n* = 1) and developmental disabilities/intellectual disabilities (*n* = 6). The age of participants ranged from 2 to 23 years. The majority of studies had parents or grandparents as informants (*n* = 11). One study included a comparison group of 17 White British parents. Two studies included children as the sole participants, one study was a case report on children and another study included a children–parent dyad as the focus of observation for an ethnographic study. The sample size ranged from 3 to 66 with a total of 266 participants.

**TABLE 2:** Characteristics of studies included in the review

Sl no	Author(s), year (country)	Title/journal	Aim (excerpt from the article)	Methodology/Method	Sample	Type of chronic conditions
1	Croot et al., 2012 (UK)	Coping strategies used by Pakistani parents living in the United Kingdom and caring for a severely disabled child. *Disability and Rehabilitation*	To explore the coping strategies of Pakistani parents living in the UK and caring for children with severe learning disabilities. To examine factors that influenced participants’ choice or ability to use the different strategies.	Qualitative design In‐depth interviews	Purposive sampling *n* = 12 (11 parents and one grandparent) (Children’s age: 4–16 years)	Developmental delay, cerebral palsy, autism and other syndromes
2	Daudji et al., 2011 (Canada)	Perceptions of disability among SA immigrant mothers of children with disabilities in Canada: Implications for rehabilitation service delivery *Disability and Rehabilitation*	To describe perceptions of disability among SA immigrant mothers of children with disabilities in a large multicultural urban centre in Ontario, Canada, and to explore how these perceptions influence rehabilitation services.	Qualitative descriptive design In‐depth face‐to‐face semi‐structured interviews	Purposive sampling *n* = 7 mothers (Children’s age: 2–14 years)	Spina bifida and congenital spinal cord injury
3	Heer et al., 2012 (UK)	The cultural context of caregiving: Qualitative accounts from SA parents who care for a child with intellectual disabilities in the UK. *Advances in Mental Health and Intellectual Disabilities*	To contribute to the development of culturally appropriate support and interventions in children’s intellectual disability services.	IPA Focused group interviews	*n* = 9 parents (5 mothers & 4 fathers) (Child’s age: 7–19 years)	Intellectual disabilities
4	Heer et al., 2015 (UK)	The experiences of British SA carers caring for a child with developmental disabilities in the UK. *Tizard Learning Disability Review*	To explore how British SA parents make sense of caregiving in the context of two different cultures	IPA In‐depth interviews	Purposive sampling *n* = 7 parents (5 mothers and 2 fathers) (Children’s age: 7–14 years)	Intellectual disabilities and developmental disabilities
5	Habib et al., 2017 (Ireland)	Pakistani mothers’ experiences of parenting a child with autism spectrum disorder (ASD) in Ireland. *Educational & Child Psychology*	To explore the parental experiences of Pakistani mothers living in Ireland who have a child with ASD	Constructivist interpretative paradigm Semi‐structured in‐depth interviews (5 face to face, 2 Skype)	Purposive sampling Seven mothers (Children’s age: 4–17 years)	Autism
6	Kelly & Kelly, 2013 (UK)	Childhood cancer‐parenting work for British Bangladeshi families during treatment: An ethnographic study *International Journal of Nursing Studies*	To detail, the day‐to‐day management experiences (including the social and cultural aspects) of cancer treatment for British Bangladeshi children and their parents.	Ethnography Participant observation in the home, community and clinical settings (22 months) Focused interviews (6 families)	Purposive sampling *n* = 15 Parent–child dyads (eight mothers and seven fathers) (Children’s age: 2 ½–12 years)	Cancer
7	Lakhanpaul et al., 2017 (UK)	A qualitative study to identify parents’ perceptions of and barriers to asthma management in children from SA and White British families *BMC Pulmonary Medicine*	To explore perceptions and experiences of asthma and asthma management in British SA and White British families, to identify barriers to optimal management and to inform culturally appropriate interventions to improve management.	Qualitative methodology Semi‐structured interviews	Purposive sampling *n* = 66 (SA carers −29 mothers, 15 fathers, five secondary carers & White British parents −17) (Children's age: Four‐12 years)	Asthma
8	Lakhanpaul et al., 2019 (UK)	Qualitative study to identify ethnicity‐specific perceptions of and barriers to asthma management in SA and white British children with asthma *BMJ Open*	To explore the perceptions and experiences of asthma in British SA and White British children using semi‐structured interviews.	Qualitative methodology Semi‐structured interviews	Purposive sampling *n* = 47 (33 SA and 14 White British children) (Children’s age: 5–12 years)	Asthma
9	Mehrotra et al., 2014 (USA)	Cultural factors impacting asthma management in Asian Indian children *Indian J* *Allergy Asthma Immunology*	To outline the cultural factors and health beliefs in the Asian Indian population which impact the care and outcome of these patients.	Case report	Purposive sampling *n* = 3 (Children at a pulmonary clinic)	Asthma
10	Mufti et al., 2015 (UK)	Pakistani children's experiences of growing up with beta‐thalassemia major *Qualitative Health Research*	To provide a rich account of children’s experiences of living with β‐TM, its management and the meanings ascribed to these experiences.	IPA Stage 1: Focused group interviews and role plays. Stage 2: Semi‐structured individual interviews	Purposive sampling *n* = 12 SA children (Children’s age: 8–12 years)	Beta‐thalassemia major
11	Ravindran & Myers, 2013 (USA)	Beliefs and practices about autism in Indian families now settled abroad: An Internet survey. *Focus on Autism and Other Developmental Disabilities*	To examine beliefs and practices about autism in Indian immigrant families having a child with autism.	Qualitative descriptive design Online questionnaire/Semi‐structured telephone interview	Purposive sampling *n* = 24 parents (21 mothers and 3 fathers) (Children’s age: 3–15 years)	Autism
12	Theara & Abbott, 2015 (UK)	Understanding the experiences of SA parents who have a child with autism *Educational & Child Psychology*	To investigate the experiences of SA parents living in the UK who have a child with autism.	GT In‐depth interviews	Purposive sampling *n* = 9 parents, (five mothers, two sets of mothers and fathers) (Children’s age: Not specified)	Autism
13	John et al., 2016 (USA)	Indian immigrant parents of children with developmental disabilities: stressors and support systems *Early Child Development and Care*	To identify key stressors and social support systems (including spousal support) among Indian immigrant families in the USA raising a child with a developmental disability. To assess the extent of parental stress and perceived quality of social support. To examine the link between parental stress and perceived quality of support	Quantitative study Online survey	*n* = 33 (25 mothers & 8 fathers) (Child’s age: 4–21 years)	Autism, cerebral palsy, Down syndrome and other developmental disabilities
14	Zechella & Raval, 2016 (USA)	Parenting children with intellectual and developmental disabilities in Asian Indian families in the United States *Journal of Child and Family Studies*	To examine unique challenges experienced by Asian Indian parents of children with IDD in U.S.A. focusing on the cultural explanations of disability, sources of stress and support, immigration experience and perceptions of the child’s future.	Qualitative Open‐ended interviews	*n* = 15 Asian Indian parents (8 mothers, 7 fathers) (Child’s age: 6–23 years)	Developmental disabilities

### 5.2 Key findings

When interpreting the findings, it is important to remember that SA ethnic groups are diverse and that the health beliefs, practices and experiences discussed may not apply to all SA immigrants.

#### 5.2.1 Theme 1: Cultural beliefs

Several beliefs existed about the causes, symptoms and management of childhood LTC (Croot et al., 2012; Daudji et al., 2011; Habib et al., 2017; Heer et al., 2012; Lakhanpaul et al., 2017; Theara & Abbott, 2015; Zechella & Raval, 2016). Family carers' beliefs significantly influenced their child's attitudes towards their illness and its management. Despite the chronic nature of their child’s condition, some family carers believed that it might 1 day be cured. A significant amount of time and money was spent on experimenting with different remedies and there were often feelings of disappointment when a child did not respond as expected (Croot et al., 2012; Daudji et al., 2011; Habib et al., 2017; Lakhanpaul et al., 2017; Theara & Abbott, 2015; Zechella & Raval, 2016). In the case of childhood asthma, family carers sometimes limited the use of inhalers unless there were symptoms because they considered asthma to be an acute condition (Lakhanpaul et al., 2017).

Extended families and SA community groups had a powerful impact on carers and their child's approach towards the LTC (Croot et al., 2012; Daudji et al., 2011; Habib et al., 2017; Heer et al., 2012; Heer et al., 2015; Kelly & Kelly, 2013; Lakhanpaul et al., 2017; Lakhanpaul et al., 2019; Mehrotra et al., 2014; Mufti et al., 2015; Ravindran & Myers, 2013; Theara & Abbott, 2015; Zechella & Raval, 2016). SA society held rigid traditional views about some of the LTC as being *bad* or *serious* or *deadly or life‐threatening or contagious* (Croot et al., 2012; Kelly & Kelly, 2013; Lakhanpaul et al., 2017; Mehrotra et al., 2014). As a result, children and their family members received negative views from SA society. They feared stigma, especially if the LTC was visible or if the treatment was evident. For example, parents felt stigmatized if their child's behaviour was disruptive in a public setting because such behaviours were viewed as culturally inappropriate and unacceptable by the local community (Croot et al., 2012). Whilst children with beta‐thalassemia major preferred to hide their infusion pumps from public view, children with asthma found using an inhaler in public to be embarrassing (Lakhanpaul et al., 2017; Lakhanpaul et al., 2019; Mufti et al., 2015). SA immigrant families preferred not to disclose the condition because of stigma. They frequently kept their children at home to reduce social interaction and delayed or minimized seeking help (Croot et al., 2012; Daudji et al., 2011; Habib et al., 2017; Heer et al., 2012; Heer et al., 2015; Kelly & Kelly, 2013; Lakhanpaul et al., 2017; Mehrotra et al., 2014; Mufti et al., 2015; Ravindran & Myers, 2013; Theara & Abbott, 2015; Zechella & Raval, 2016).

Regardless of the type of LTC, SA family carers provided several religious explanations for the cause of their child's condition. Many believed that the LTC was the consequence of God’s will and associated it with karma (a concept that one's past actions influence their destiny) (Croot et al., 2012; Lakhanpaul et al., 2017; Mufti et al., 2015; Ravindran & Myers, 2013). Others associated the LTC with a curse from ancestors or a punishment from God or a test from God (Croot et al., 2012; Zechella & Raval, 2016). Conversely, some parents believed that children with developmental disabilities were a gift from God; they viewed parenting as an opportunity for personal development or discovery of a greater purpose in life (Croot et al., 2012; Zechella & Raval, 2016). Many parents, even with a clear understanding of the medical cause of their child's condition, explored the spiritual meaning of their experiences and identified it as an important coping resource (Croot et al., 2012; Daudji et al., 2011; Habib et al., 2017; Heer et al., 2012; Heer et al., 2015; Lakhanpaul et al., 2017; Lakhanpaul et al., 2019; Mehrotra et al., 2014; Mufti et al., 2015; Ravindran & Myers, 2013; Theara & Abbott, 2015; Zechella & Raval, 2016).

The review provided insights into beliefs specific to certain conditions. SA family carers related the cause of their child's developmental disorders to non‐biomedical factors such as vaccine injury, poor medical care during pregnancy and ineffective parenting (Croot et al., 2012; Heer et al., 2012; Kelly & Kelly, 2013; Ravindran & Myers, 2013). Going out during an eclipse whilst pregnant has been linked to causing developmental disorders by some Indian families because of the belief that it would result in the accumulation of negative energy (Zechella & Raval, 2016). Smoking during pregnancy was cited as the cause of asthma in one study (Lakhanpaul et al., 2017). Mothers were blamed for their child's illness based on such beliefs. SA parents believed that asthma is triggered because of a hot–cold imbalance. According to the hot–cold theory, illness occurs when the body’s equilibrium is disrupted by being excessively *hot* or *cold* (Roodaki et al., 2018). SA immigrants expected asthma flare‐ups if their children had physically cold foods or foods with a cold base (banana, yogurt, grapes, etc.), had a cold shower or were improperly dressed. Many of them held a misconception around activities as a trigger for asthma exacerbation which resulted in the imposition of restrictions on a child's activities (Lakhanpaul et al., 2017; Lakhanpaul et al., 2019; Mehrotra et al., 2014).

#### 5.2.2 Theme 2: Religious, spiritual and complementary and alternative medicine practices

SA family carers engaged in various religious and spiritual practices to help them cope with their child’s LTC. Offering prayers, visiting holy places, checking horoscopes, making oaths, wearing special stones and amulets, fasting and consulting spiritual healers were some examples (Croot et al., 2012; Daudji et al., 2011; Habib et al., 2017; Heer et al., 2012; Heer et al., 2015; Kelly & Kelly, 2013; Lakhanpaul et al., 2017; Lakhanpaul et al., 2019; Mehrotra et al., 2014; Mufti et al., 2015; Ravindran & Myers, 2013; Theara & Abbott, 2015; Zechella & Raval, 2016).

SA family carers used Complementary and Alternative Medicine (CAM) for their children believing that it had fewer side effects but was as effective as Western medicine (Daudji et al., 2011; Habib et al., 2017; Heer et al., 2015; Lakhanpaul et al., 2017; Lakhanpaul et al., 2019; Mehrotra et al., 2014; Mufti et al., 2015; Ravindran & Myers, 2013; Theara & Abbott, 2015; Zechella & Raval, 2016). Ravindran and Myers (2013) found that many Indian immigrant parents of children with autism adopted a combination of modern treatments and traditional Indian treatment methods (e.g., used Homoeopathy, Ayurveda, Yoga, music therapies, etc.) to ensure the best possible care. Although CAM was used as an adjunct treatment in most childhood LTC, these practices were especially popular in the case of childhood asthma as parents were concerned about medication side effects, predominantly steroid addiction (Mehrotra et al., 2014). Parents were frequently pressured by their extended older family members to use traditional medicine or other natural treatments for a cure (Heer et al., 2015; Mehrotra et al., 2014).

#### 5.2.3 Theme 3: Care and support of the child

Although mothers were typically the primary caregivers in most SA immigrant families, they received enormous support from their spouses in childcare (Croot et al., 2012; Daudji et al., 2011). Mothers stayed at home or worked part‐time to care for their children. Caring for their children with LTC was reported as physically and emotionally exhausting. They made personal sacrifices with respect to paid employment or self‐care, prioritizing the well‐being of the child and the family. Mothers balanced multiple roles ranging from being a full‐time caregiver to that of an advocate for their child (Croot et al., 2012; Daudji et al., 2011; Heer et al., 2015; Kelly & Kelly, 2013; Lakhanpaul et al., 2017; Lakhanpaul et al., 2019; Zechella & Raval, 2016). They encountered challenges with HCP and school staff to ensure optimal care for their children (Croot et al., 2012; Habib et al., 2017; Kelly & Kelly, 2013; Lakhanpaul et al., 2017; Ravindran & Myers, 2013; Zechella & Raval, 2016).

Schools were one of the main sources of support and school services were widely acknowledged as important to the child’s well‐being (Croot et al., 2012; Habib et al., 2017; Lakhanpaul et al., 2019; Mufti et al., 2015; Ravindran & Myers, 2013). For children with developmental disorders, some parents found school valuable because it provided them with time away from their children and others found their children learnt new skills (Croot et al., 2012). In contrast, participants also expressed their dissatisfaction with the school system for a variety of reasons, including peer bullying, discrimination from school staff, lack of resources including adequately trained staff, administrative bureaucracy and poor services (Croot et al., 2012; Habib et al., 2017; Mufti et al., 2015; Ravindran & Myers, 2013; Zechella & Raval, 2016).

The findings from the review on informal support systems were mixed. Children described their parents as an enormous source of support throughout their illness journey (Lakhanpaul et al., 2019; Mufti et al., 2015). They also reported negative consequences related to parental care that led to overindulgence and overprotection, which reinforced children’s perception of being different (Mufti et al., 2015).

Lack of extended family support in the host country was a major concern (Croot et al., 2012; Habib et al., 2017; Ravindran & Myers, 2013; Zechella & Raval, 2016). Some of the studies of participants with asthma and cancer found that carers relied substantially on family and relatives for support. However, studies reporting on children with developmental disabilities revealed that they received little assistance from their extended family network (Croot et al., 2012). Parents with children who had physical and mental disabilities experienced loneliness. They struggled even if they had their extended families in the host country because they did not accept these children due to stigma or challenges related to providing care (Croot et al., 2012). According to a study conducted by John et al. (2016) among Indian immigrant parents of children with developmental disabilities, a negative link was identified between the parents' reported quality of social support and their stress levels. In this study, the most important source of support for the participants was their spouse, followed by support groups and friends, with HCP at the bottom of the list.

Family caregivers and children expressed diverse feelings about the health‐care services they received. HCP were appreciated for the informational and emotional support provided which helped them cope with their challenges. Simultaneously, negative experiences with HCP included a range of areas: lack of proper care during pregnancy resulting in child's illness, delay in getting a diagnosis, not being listened to, showing culture blaming and a discriminatory attitude, poor service quality, etc (Croot et al., 2012; Heer et al., 2015; Kelly & Kelly, 2013; Lakhanpaul et al., 2017; Lakhanpaul et al., 2019). In the study by Lakhanpaul et al. (2019), some children expressed their concern about feeling ‘left out’ during doctors' consultations, which they highlighted as more family carer‐centred discussions. Additionally, one of the significant barriers to building meaningful relationships with HCP was limited English proficiency which also restricted the family carers from seeking timely care (Croot et al., 2012; Heer et al., 2015; Lakhanpaul et al., 2017; Mehrotra et al., 2014).

Many studies found that health service uptake was low among Indian immigrant parents with certain LTC such as developmental and intellectual disabilities (Croot et al., 2012; Heer et al., 2012). Even if they used services, they accessed generic welfare services such as daycare, rather than specialist options and long‐term respite services. According to the studies, one possible explanation is that most SA children grew up in two‐parent families, and children from such households were less likely to use formal services. Poor knowledge about the health‐care system such as availability and accessibility of services was yet another key barrier to health service utilization. Barriers in accessing health services included challenges with appointment accessibility, long waiting times, after‐hours access, language barriers, lack of trust in the host health‐care system, etc (Croot et al., 2012; Heer et al., 2015; Lakhanpaul et al., 2017; Mehrotra et al., 2014).

## 6 DISCUSSION

The findings of the review suggest that SA immigrant children's and their family carers' interpretations of LTC are predominantly based on their sociocultural influences and often contrasts with the biomedical models (Heer et al., 2015; Lakhanpaul et al., 2017; Ravindran & Myers, 2013; Theara & Abbott, 2015). For example, the biomedical model shapes people’s perceptions of autism by portraying disability as a deficit and children with disabilities as those who need to be fixed (Bagatell, 2010). Social models, on the other hand, argue that disability is a socially constructed concept, with the sociocultural context having a substantial impact on how children, their families and the wider society view and manage disability (Theara & Abbott, 2015).

The findings, consistent with the social models, reveal a complex interaction of various sociocultural factors such as cultural beliefs, religious, spiritual and CAM practices and migration influencing SA immigrants’ experiences of childhood LTC (Heer et al., 2012; Heer et al., 2015; Lakhanpaul et al., 2017; Mehrotra et al., 2014; Ravindran & Myers, 2013; Theara & Abbott, 2015). This is commensurate with Ahmed et al.'s (2018) systematic review of randomized controlled trials on SA's asthma self‐management behaviour. The researchers found out that interventions delivered to SAs in their host country were less effective than those provided to South Asia’s indigenous population. The findings, therefore, emphasize the need to explore the impact of sociocultural factors, which are dynamic and constantly shaped by place and time. CFCC enables HCPs to examine, build on and incorporate these sociocultural factors into management plans for better compliance. However, in line with other studies, our findings show a significant disparity between what CFCC should be and what occurs in practice (Gerlach & Varcoe, 2020; Watt et al., 2011).

The findings reflect the attitude of SA immigrants who may choose to keep their traditional beliefs, practices and experiences to themselves without disclosing it to HCP. For example, Mehrotra et al. (2014) highlighted the use of CAM as the first‐line treatment strategy for childhood asthma by many Indian parents without the awareness of their physician. Some of the factors identified that restricted these parents from disclosing their beliefs and practices included lack of confidence in the host health‐care system, fear of being blamed for their beliefs and fear of discrimination. These findings are consistent with previous research on health disparities among minority groups. For example, similar attitudes were shown by Hispanic parents who hesitated to share their folk‐related practices on common childhood illnesses with HCP (Acorda et al., 2020). On the other hand, SA immigrants may also assume that HCP share the same tacit understanding as their own. The findings, therefore, reiterate the call for HCP to be proactive by asking culturally relevant questions to elicit the SA immigrant children's and family carers' cultural needs and expectations (Englund & Rydström, 2012).

This review has highlighted the interconnectedness of culture and health and the importance of HCP to work in a culturally safe manner (Curtis et al., 2019). Curtis et al. (2019) describe, ‘Cultural safety is about acknowledging the barriers to clinical effectiveness arising from the inherent power imbalance between provider and patient (p.13). Culturally safe HCP empower SA immigrant families to be involved in their own care and facilitate the development of a mutually agreed realistic care plan (Lakhanpaul et al., 2014). For instance, an awareness of the close‐knit family and community structure of SA families will allow HCP to consider the involvement of extended family members and community experts as a part of the decision‐making process. Simultaneously, HCP should be aware that the extended family and community can be a source of both support (informational, instrumental and emotional support) and stress (contradictory views about treatment options and stigmatizing attitude) for the SA family carers and children. Therefore, the exploration of complex family dynamics becomes crucial when working with these families (Daudji et al., 2011; Heer et al., 2015; Theara & Abbott, 2015).

Additionally, HCP must realize that cultural safety cannot be attained through the application of generalized cultural assumptions (Curtis et al., 2019; Englund & Rydström, 2012; Heer et al., 2015). Although traditional values, beliefs and cultural norms play a key role in shaping the health behaviours of SA immigrant children and their families, each family differs in their degree of acceptance of these traditional norms. Factors such as education, socio‐economic status, religion, degree of acculturation, length of stay in the host country and English proficiency have a significant impact on an SA immigrants’ health beliefs and practices. HCP should work on the principle that each member of an ethnic group has their own distinct culture (Heer et al., 2015). These findings are consistent with the results from Ahmed et al.'s (2018) systematic review which identified a lack of cultural awareness and a failure to recognize the ethnoreligious heterogeneity in the SA community as the primary reasons for the failure of targeted interventions aimed at improving asthma outcomes. Therefore, the challenge for clinicians, researchers and policymakers is to develop culturally safe child‐ and family‐centred interventions to address the specific needs of SA immigrant children.

## 7 LIMITATIONS

The review included studies published only in English. Therefore, not all studies on this topic may have been retrieved. A major portion of the studies focused on one group of LTC – developmental disorders. Hence, the findings are more probably a reflection of the beliefs, practices and experiences of SA immigrant families who have children with developmental disorders. Moreover, the findings might be a more accurate reflection of family carers’ perspectives than that of the children's because most studies listened to the voices of family carers. Given the exploratory nature of this review, differences in beliefs, practices and experiences between different LTCs, sub‐ethnic SA groups and host countries have not been adequately explored. The review also did not distinguish between the experiences of newly migrated and already established SA immigrants. This is significant because an immigrant family’s length of stay in a country may influence their health‐care choices. All these limitations should be considered when interpreting the findings of the review and when designing future studies.

## 8 IMPLICATIONS FOR HEALTH‐CARE PRACTICE AND RESEARCH

The review demonstrates the significant influence of the sociocultural context in reinforcing the meanings ascribed to childhood LTC management among SA immigrants. Hence, HCP should consider the collectivist cultural nature of SA immigrants when planning interventions for this ethnically diverse population. This could be accomplished by integrating cultural assessments into treatment care pathways. The findings have implications for health‐care delivery in general since they emphasize the importance of cultural safety.

Longitudinal studies on how SA family caregivers gain the expertise to manage their child’s illness might help determine the best strategies to support these families. The methods for researching experiences of a SA immigrant child with an LTC are dominated by face‐to‐face interviews. A broader population can be reached by offering alternative data collection options such as Internet‐based research methods alongside the traditional ones. This approach will allow eliciting the views of those who choose to remain anonymous. The current review showed a dominance of family carer‐oriented studies despite the increasing emphasis on giving voice to children in all the matters affecting them. Therefore, more child‐centred research using child‐friendly data collection techniques must be undertaken to obtain a unique and detailed understanding of the experiences of these children. Additionally, future studies should include a wide range of LTC as well as SA immigrant children from non‐English speaking countries. Furthermore, future studies should focus on analysing the health beliefs and practices of individuals in each SA country rather than the entire SA population.

## 9 CONCLUSION

The review provides insights into the disparities in expectations around the management of childhood LTC that exist between SA immigrant families and HCP, resulting in misunderstanding and strain in their relationships. HCP should use a combination of culturally safe management strategies and a nuanced approach to educational initiatives on the biomedical aspects of various LTC to effectively engage SA immigrant families with health services. The review also uncovers the burden of care for SA family caregivers in the host country and emphasizes the necessity of additional support measures. The clinicians, researchers and policymakers can use the findings to better understand and support the needs of SA immigrant families who have children with LTC.

## CONFLICT OF INTEREST

No conflict of interest has been declared by the authors.

## AUTHOR CONTRIBUTIONS

Indu Sudarsan completed the literature search, reviewed the studies and prepared the manuscript. All the other authors made substantial contributions to the conception and design of the study. Selection and quality appraisal of studies, data extraction: Karen Hoare as the second reviewer, Nicolette Sheridan as the third reviewer and Jennifer Roberts as the fourth reviewer.

All authors have met all the following criteria:

Have made substantial contributions to conception and design or acquisition of data or analysis and interpretation of data.

1. Been involved in drafting the manuscript or revising it critically for important intellectual content.
2. Given final approval of the version to be published. Each author has participated sufficiently in the work to take public responsibility for appropriate portions of the content.
3. Agreed to be accountable for all aspects of the work in ensuring that questions related to the accuracy or integrity of any part of the work are appropriately investigated and resolved.

### PEER REVIEW

The peer review history for this article is available at https://publons.com/publon/10.1111/jan.15217.

## Supporting information


**Table S1.** Critical search terms and the expanded terms.

**Table S2.** List of full‐text screened articles.

**Table S3.** CASP Qualitative Checklist.

**Table S4.** JBI Critical Appraisal Checklist for analytical cross‐sectional studies.

## Data Availability

The data that support the findings of this study are available from the corresponding author upon reasonable request.

## References

[jan15217-bib-0001] Acorda, D. E. , DesOrmeaux, C. N. , Rozmus, C. L. , & Engebretson, J. C. (2020). Hispanic parental beliefs and practices in the management of common childhood illnesses: A review of the literature. Journal of Transcultural Nursing, 31(5), 502–518. 10.1177/1043659620935970 32567512

[jan15217-bib-0002] Ahmed, S. , Steed, L. , Harris, K. , Taylor, S. J. , & Pinnock, H. (2018). Interventions to enhance the adoption of asthma self‐management behavior in the SA and African American population: A systematic review. NPJ Primary Care Respiratory Medicine, 28(1), 1–20. https://doi.org/10.1038/s41533-017-0070-6 PMC5814446 29449558

[jan15217-bib-0003] Bagatell, N. (2010). From cure to community: Transforming notions of autism [special issue]. Ethos, 38, 33–55.

[jan15217-bib-0004] Batra, M. , Gupta, S. , & Erbas, B. (2019). Oral health beliefs, attitudes, and practices of SA migrants: A systematic review. International Journal of Environmental Research and Public Health, 16(11), 1952. 10.3390/ijerph16111952 PMC6603871 31159420

[jan15217-bib-0005] Benchimol, E. I. , Manuel, D. G. , To, T. , Mack, D. R. , Nguyen, G. C. , Gommerman, J. L. , Croitoru, K. , Mojaverian, N. , Wang, X. , Quach, P. , & Guttmann, A. (2015). Asthma, type 1 and type 2 diabetes mellitus, and inflammatory bowel disease amongst SA immigrants to Canada and their children: A population‐based cohort study. PLoS One, 10(4), e0123599. 10.1371/journal.pone.0123599 25849480 PMC4388348

[jan15217-bib-0006] Berger, P. L. , & Luckmann, T. (1991). The social construction of reality: A treatise in the sociology of knowledge. Open Road Media.

[jan15217-bib-0007] Burr, V. (2003). Social constructionism. Psychology Press.

[jan15217-bib-0008] Burr, V. (2015). An Introduction to social constructionism. Routledge.

[jan15217-bib-0009] Conrad, P. , & Barker, K. K. (2010). The social construction of illness: Key insights and policy implications. Journal of Health and Social Behavior, 51(1_suppl), S67–S79. 10.1177/0022146510383495 20943584

[jan15217-bib-0010] Critical Appraisal Skills Programme . (2018). CASP checklist. 10 questions to help you make sense of a qualitative research https://casp‐uk.net/wp‐content/uploads/2018/01/CASP‐Qualitative‐Checklist‐2018.pdf

[jan15217-bib-0011] Croot, E. , Grant, G. , Mathers, N. , & Cooper, C. (2012). Coping strategies used by Pakistani parents living in the United Kingdom and caring for a severely disabled child. Disability and Rehabilitation, 34(18), 1540–1549. 10.3109/09638288.2011.650310 22304691

[jan15217-bib-0012] Curtis, E. , Jones, R. , Tipene‐Leech, D. , Walker, C. , Loring, B. , Paine, S. , & Reid, P. (2019). Why cultural safety rather than cultural competency is required to achieve health equity: A literature review and recommended definition. International Journal for Equity in Health, 18(174), 1–17. 31727076 10.1186/s12939-019-1082-3 PMC6857221

[jan15217-bib-0013] Daudji, A. , Eby, S. , Foo, T. , Ladak, F. , Sinclair, C. , Landry, M. D. , Moody, K. , & Gibson, B. E. (2011). Perceptions of disability among SA immigrant mothers of children with disabilities in Canada: Implications for rehabilitation service delivery. Disability and Rehabilitation, 33(6), 511–521. 20597810 10.3109/09638288.2010.498549

[jan15217-bib-0014] Didham, R. (2010). Future potential and the invisible diaspora: New Zealand and SA diasporas. Asia New Zealand Foundation https://www.asianz.org.nz/assets/Uploads/Future‐Potential‐and‐the‐Invisible‐Diaspora‐New‐Zealand‐and‐South‐Asia‐diaspora.pdf

[jan15217-bib-0015] Englund, A. D. , & Rydström, I. (2012). I have to turn myself inside out. Clinical Nursing Research, 21(2), 224–242. 10.1177/1054773812438915 22473272

[jan15217-bib-0016] Ford, K. , Dickinson, A. , Water, T. , Campbell, S. , Bray, L. , & Carter, B. (2018). Child centred care: Challenging assumptions and repositioning children and young people. Journal of Pediatric Nursing, 43, e39–e43. 10.1016/j.pedn.2018.08.012 30172421

[jan15217-bib-0017] Gergen, K. J. (2015). An invitation to social construction (3rd ed.). Sage.

[jan15217-bib-0018] Gerlach, A. , & Varcoe, C. (2020). Orienting child‐ and family‐centered care toward equity. Journal of Child Health Care, 25(3), 457–467. 10.1177/1367493520953354 32853028

[jan15217-bib-0019] Gupta, V. B. (2010). Impact of culture on healthcare seeking behavior of Asian Indians. Journal of Cultural Diversity, 17(1), 13–19. 20397569

[jan15217-bib-0020] Habib, S. , Prendeville, P. , Abdussabur, A. , & Kinsella, W. (2017). Pakistani mother's experiences of parenting a child with autism spectrum disorder (ASD) in Ireland. Educational & Child Psychology, 34(2), 67–79.

[jan15217-bib-0021] Heer, K. , Larkin, M. , Burchess, I. , & Rose, J. (2012). The cultural context of caregiving: Qualitative accounts from SA parents who care for a child with intellectual disabilities in the UK. Advances in Mental Health and Intellectual Disabilities, 6(4), 179–191. 10.1108/20441281211236580

[jan15217-bib-0022] Heer, K. , Larkin, M. , & Rose, J. (2015). The experiences of British SA carers caring for a child with developmental disabilities in the UK. Tizard Learning Disability Review, 20(4), 228–238. 10.1108/tldr-12-2014-0044

[jan15217-bib-0023] International Organisation for Migration . (2019). World migration report 2020. https://publications.iom.int/system/files/pdf/wmr_2020.pdf

[jan15217-bib-0024] Joanna Brigg's Institute . (2020). Critical appraisal tool. https://jbi.global/critical‐appraisal‐tools

[jan15217-bib-0025] John, A. , Bower, K. , & McCullough, S. (2016). Indian immigrant parents of children with developmental disabilities: Stressors and support systems. Early Child Development and Care, 186(10), 1594–1603. 10.1080/03004430.2015.1116297

[jan15217-bib-0026] Kelly, P. , & Kelly, D. (2013). Childhood cancer‐parenting work for British Bangladeshi families during treatment: An ethnographic study. International Journal of Nursing Studies, 50(7), 933–944. 10.1016/j.ijnurstu.2012.11.004 23218019

[jan15217-bib-0027] Knafl, K. , & Whittemore, R. (2017). Top 10 tips for undertaking synthesis research. Research in Nursing & Health, 40(3), 189–193. 10.1002/nur.21790 28267870

[jan15217-bib-0028] Lakhanpaul, M. , Bird, D. , Manikam, L. , Culley, L. , Perkins, G. , Hudson, N. , Wilson, J. , & Johnson, M. (2014). A systematic review of explanatory factors of barriers and facilitators to improving asthma management in SA children. BMC Public Health, 14(1), 1–11. 10.1186/1471-2458-14-403 24767303 PMC4032170

[jan15217-bib-0029] Lakhanpaul, M. , Culley, L. , Huq, T. , Bird, D. , Hudson, N. , Robertson, N. , McFeeters, M. , Manikam, L. , Johal, N. , Hamlyn‐Williams, C. , & Johnson, M. R. (2019). Qualitative study to identify ethnicity‐specific perceptions of and barriers to asthma management in SA and white British children with asthma. BMJ Open, 9(2), e024545. 10.1136/bmjopen-2018-024545 PMC6411253 30782908

[jan15217-bib-0030] Lakhanpaul, M. , Culley, L. , Robertson, N. , Alexander, E. C. , Bird, D. , Hudson, N. , Johal, N. , McFeeters, M. , Hamlyn‐Williams, C. , Manikam, L. , Boo, Y. Y. , Lakhanpaul, M. , & Johnson, M. R. (2020). A structured collaborative approach to intervention design using a modified intervention mapping approach: A case study using the management and interventions for asthma (MIA) project for SA children. BMC Medical Research Methodology, 20(1), 1–16. 10.1186/s12874-020-01148-y PMC7607819 33138784

[jan15217-bib-0031] Lakhanpaul, M. , Culley, L. , Robertson, N. , Bird, D. , Hudson, N. , Johal, N. , McFeeters, M. , Angell, E. , Hamlyn‐Williams, C. , Abbas, N. , Manikam, L. , & Johnson, M. (2017). A qualitative study to identify parents' perceptions of and barriers to asthma management in children from SA and white British families. BMC Pulmonary Medicine, 17(1), 1–12. 10.1186/s12890-017-0464-9 28931381 PMC5607610

[jan15217-bib-0032] Liu, J. J. , Davidson, E. , Bhopal, R. , White, M. , Johnson, M. , Netto, G. , & Sheikh, A. (2016). Adapting health promotion interventions for ethnic minority groups: A qualitative study. Health Promotion International, 31(2), 325–334. 10.1093/heapro/dau105 25561680

[jan15217-bib-0033] Mann, M. (2014). South Asia's modern history: Thematic perspectives. Routledge.

[jan15217-bib-0034] Mehrotra, N. , Ramagopal, M. , & Dodani, S. (2014). Cultural factors impacting asthma management in Asian Indian children. Indian Journal of Allergy, Asthma and Immunology, 28(2), 63. 10.4103/0972-6691.140761

[jan15217-bib-0035] Mehta, S. (2012). Health needs assessment of Asian people living in the Auckland region. https://countiesmanukau.health.nz/assets/About‐CMH/Performance‐and‐planning/health‐status/2012‐health‐needs‐of‐asian‐people.pdf

[jan15217-bib-0036] Moher, D. , Liberati, A. , Tetzlaff, J. , Altman, D. G. , & Prisma Group . (2009). Preferred reporting items for systematic reviews and meta‐analyses: The PRISMA statement. Annals of Internal Medicine, 151(4), 264. 10.7326/0003-4819-151-4-200908180-00135 19622511

[jan15217-bib-0037] Mufti, G. , Towell, T. , & Cartwright, T. (2015). Pakistani children's experiences of growing up with beta‐thalassemia major. Qualitative Health Research, 25(3), 386–396. 10.1177/1049732314552663 25249550

[jan15217-bib-0038] Ramaswamy, P. , Mathew Joseph, N. , & Wang, J. (2019). Health beliefs regarding cardiovascular disease risk and risk reduction in south Asian immigrants: An integrative review. Journal of Transcultural Nursing, 31(1), 76–86. 10.1177/1043659619839114 30957667

[jan15217-bib-0039] Ravindran, N. , & Myers, B. J. (2013). Beliefs and practices regarding autism in Indian families now settled abroad. Focus on Autism and Other Developmental Disabilities, 28(1), 44–53. 10.1177/1088357612458970

[jan15217-bib-0040] Roodaki, M. , Faridi, P. , Abolhasanzadeh, Z. , & Karimzadeh, I. (2018). Hot and cold: An old theory with modern applications. Trends in Pharmaceutical Sciences, 4(1), 59–82.

[jan15217-bib-0041] Theara, G. , & Abbott, D. W. (2015). Understanding the experiences of SA parents who have a child with autsim. Educational and Child Psychology, 32(2), 47–56.

[jan15217-bib-0042] Watt, L. , Dix, D. , Gulati, S. , Sung, L. , Klaassen, R. J. , Shaw, N. T. , & Klassen, A. F. (2011). Family‐centred care: A qualitative study of Chinese and SA immigrant parents' experiences of care in paediatric oncology. Child: Care, Health and Development, 39(2), 185–193. 10.1111/j.1365-2214.2011.01342.x 22066491

[jan15217-bib-0043] Whittemore, R. , & Knafl, K. (2005). The integrative review: Updated methodology. Journal of Advanced Nursing, 52(5), 546–553. 10.1111/j.1365-2648.2005.03621.x 16268861

[jan15217-bib-0044] World Health Organization . (2014). Adolescence: A period needing special attention ‐ recognizing‐adolescence. https://apps.who.int/adolescent/second‐decade/section2/page1/recognizing‐adolescence.html

[jan15217-bib-0045] Zechella, A. N. , & Raval, V. V. (2016). Parenting children with intellectual and developmental disabilities in Asian Indian families in the United States. Journal of Child and Family Studies, 25(4), 1295–1309. 10.1007/s10826-015-0285-5

