# Supplementary material for: South Asian immigrants’ and their family carers’ beliefs, practices and experiences of childhood long‐term conditions: An integrative review
Source: J Adv Nurs. 2022 Mar 14;78(7):1897–908. doi: 10.1111/jan.15217 (PMC9314788; doi:10.1111/jan.15217)
Supplement: Supplementary file 1 — Table S1. Critical search terms and the expanded terms. [file JAN-78-1897-s003.docx]

**Supplemental file 1: Critical search terms and the expanded terms**

| **The searched term** | **The expanded terms** |
| --- | --- |
| chronic* | asthma* OR wheez* OR resp* OR eczema* OR cancer* OR diabetes* OR autism* OR development* OR congenital* OR illness* OR sickness* OR health* OR "long term*" |
| "South Asia*" | India* OR Pakistan* OR Bangladesh* OR Punjab* OR Gujarat* |
| child* | pediatric* OR paediatric* OR adolesc* OR teen* OR "young adult*" |
| migrant* | immigrant* |
| "care giver*" | carer* OR caregiver* OR parent* OR famil* |
