# Supplementary material for: South Asian immigrants’ and their family carers’ beliefs, practices and experiences of childhood long‐term conditions: An integrative review
Source: J Adv Nurs. 2022 Mar 14;78(7):1897–908. doi: 10.1111/jan.15217 (PMC9314788; doi:10.1111/jan.15217)
Supplement: Supplementary file 2 — Table S2. List of full‐text screened articles. [file JAN-78-1897-s001.docx]

**Supplemental file 2: List of full text screened articles**

| **Sl No** | **Article title** | **Author(s)/year/**  **country** | **Decision** | **Explanation** | **Source** |
| --- | --- | --- | --- | --- | --- |
| 1 | Beliefs and practices regarding autism in Indian families now settled abroad: An internet survey | Ravindran and Myers/ 2012/USA | Included | The internet survey examined the beliefs and practices of 24 immigrant parents (from USA, Canada and Kuwait) having a child with autism | Database searching |
| 2 | The experiences of British South Asian carers caring for a child with developmental disabilities in the UK | Heer et al/2015/UK | Included | The qualitative study explored the experiences of seven British South Asian parents (five mothers and two fathers) caring for a child with developmental disabilities using interpretative phenomenological analysis | Database searching |
| 3 | Childhood cancer-parenting work for British Bangladeshi families during treatment: An ethnographic study | Kelly and Kelly/2012/UK | Included | The ethnographic study detailed the day-to-day management experiences (including the social and cultural aspects) of cancer treatment for British Bangladeshi children and their parents. | Database searching |
| 4 | Family-centred care: a qualitative study of Chinese and South Asian immigrant parents’ experiences of care in paediatric oncology | Watt et al/2012/Canada | Excluded | The constructivist grounded theory study described Chinese and South Asian immigrant parents’ experiences of family centred care in paediatric oncology settings in Canada.  Findings specific to SA immigrant parents could not be extracted. | Database searching |
| 5 | Immigrant to Canada, newcomer to childhood cancer: A qualitative study of challenges faced by immigrant parents | Klassen et al/2012/Canada | Excluded | The constructivist grounded theory study explored any special challenges faced by Chinese and South Asian immigrant parents of children with cancer and to identify supportive factors.  Findings specific to SA immigrant parents could not be extracted. | Database searching |
| 6 | Communication and language challenges experienced by Chinese and South Asian immigrant parents of children with cancer in Canada: Implications for Health Services Delivery | Gulati et al/2012/Canada | Excluded | The constructivist grounded theory study explored the role of communication and language in the healthcare experiences of immigrant parents of children with cancer living in Canada.  Findings specific to SA immigrant parents could not be extracted. | Database searching |
| **7** | Coping strategies used by Pakistani parents living in the United Kingdom and caring for a severely disabled child. | Croot et al/2012/UK | Included | The qualitative study explored the coping strategies of Pakistani parents living in the UK and caring for children with severe learning disabilities. It examined factors that influenced participants’ choice or ability to use the different strategies. | Database searching |
| 8 | Parenting children with intellectual and developmental disabilities in Asian Indian Families in the United States | Zechella & Raval/2016/USA | Included | The qualitative study described unique experiences and challenges of Asian Indian immigrant parents (15 Asian Indian parents (8 mothers, 7 fathers)) of children with intellectual and developmental disabilities (IDD) in USA. | Database searching |
| 9 | Indian immigrant parents of children with developmental disabilities: stressors and support systems | John et al/2016/USA | Included | The study examined the stressors and perceived quality of social support among Indian immigrant families of children and adolescents with a developmental disability in the USA. | Database searching |
| 10 | Pakistani mothers’ experiences of parenting a child with autism spectrum disorder (ASD) in Ireland | Habib et al/2017/Ireland | Included | The qualitative study used a constructivist interpretative paradigm and a culturally sensitive approach to explore the parental experiences of Pakistani mothers (n=7) living in Ireland who have a child with autism spectrum disorder (ASD) | Database searching |
| 11 | Perceptions of disability among south Asian immigrant mothers of children with disabilities in Canada: implications for rehabilitation service delivery | Daudji et al/2011/Canada | Included | The descriptive qualitative study described perceptions of disability among South Asian immigrant mothers (n=5) of children with disabilities in a large multicultural urban centre in Ontario, Canada, and explored how these perceptions influence rehabilitation services. | Database searching |
| 12 | Cultural beliefs and coping strategies related to childhood cancer: The perceptions of South Asian immigrant parents in Canada | Banerjee et al/2011/Canada | Excluded | The constructivist grounded theory study aimed to describe cultural beliefs and coping strategies related to dealing with childhood cancer identified through a qualitative study of the caregiving experiences of first-generation South Asian immigrant parents of children with cancer.  Secondary data analysis | Database searching |
| 13 | Understanding the experiences of South Asian parents who have a child with autism | Theara & Abbott/2015/UK | Included | The grounded theory study investigated the experiences of South Asian parents (Nine parents, five mothers, two sets of mothers and fathers) living in the UK who have a child with autism. | Reference tracking |
| 14 | The cultural context of caregiving: qualitative accounts from South Asian parents who care for a child with intellectual disabilities in the UK | Heer et al/2012/UK | Included | Through a hermeneutic and phenomenological approach, study set out to develop a culturally sensitive account of Sikh and Muslim parents’ experiences of caring for a child with intellectual disabilities. | Reference tracking |
| 15 | Pakistani children’s experiences of growing up with Beta-Thalassemia Major | Mufti et al/2015/UK | Included | Through Interpretive phenomenological analytic approach, the study provided a rich account of children’s experiences of living with β-TM, its management, and the meanings ascribed to these experiences. | Reference tracking |
| 16 | The impact of thalassemia on Southeast Asian and Asian Indian families in the United States: A qualitative study | Liem et al/2011/USA | Excluded | The qualitative described the challenges including sociocultural and socioeconomic barriers, faced by an urban immigrant population in the United States by Thalassemia major  Findings specific to Asian Indian immigrant parents could not be extracted. | Reference tracking |
| 17 | A qualitative study to identify parents’ perceptions of and barriers to asthma management in children from South Asian and White British families | Lakhanpaul et al//2017/UK | Included | The qualitative study explored perceptions and experiences of asthma and asthma management in British South Asian and White British families, identified barriers to optimal management and informed culturally appropriate interventions to improve management. | Reference tracking |
| 18 | Qualitative study to identify ethnicity specific perceptions of and barriers to asthma management in South Asian and White British children with asthma | Lakhanpaul et al//2019/UK | Included | The qualitative study explored the perceptions and experiences of asthma in British South Asian children using semi-structured interviews. | Reference tracking |
| 19 | Cultural factors impacting asthma management in Asian Indian children | Mehrotra et al/2014/USA | Included | The case report outlined the cultural factors and health beliefs in the Asian Indian population which impacted the care and outcome of these patients. | Reference tracking |
