# Supplementary material for: South Asian immigrants’ and their family carers’ beliefs, practices and experiences of childhood long‐term conditions: An integrative review
Source: J Adv Nurs. 2022 Mar 14;78(7):1897–908. doi: 10.1111/jan.15217 (PMC9314788; doi:10.1111/jan.15217)
Supplement: Supplementary file 3 — Table S3. CASP Qualitative Checklist. [file JAN-78-1897-s002.docx]

**Supplemental file 3: CASP Qualitative Checklist**

| Sl No | Assessment questions | Lakhanpaul et al., 2019 | Lakhanpaul et al., 2017 | Mehrotra et al., 2014 | Kelly & Kelly, 2012 | Mufti et al, 2015 | Ravindran & Myers, 2012 | Habib et al.,2017 | Theara & Abbott, 2015 | Zechella & Raval, 2016 | Croot et al, 2012 | Daudji et al., 2011 | Heer et al., 2015 | Heer et al., 2012 |
| --- | --- | --- | --- | --- | --- | --- | --- | --- | --- | --- | --- | --- | --- | --- |
| 1 | Was there a clear statement of the aims of the research? | Yes | Yes | Yes | Yes | Yes | Yes | Yes | Yes | Yes | Yes | Yes | Yes | Yes |
| 2 | Is a qualitative methodology appropriate? | Yes | Yes | Yes | Yes | Yes | Yes | Yes | Yes | Yes | Yes | Yes | Yes | Yes |
| 3 | Was the research design appropriate to address the aims of the research? | Yes | Yes | Yes | Yes | Yes | Yes | Yes | Yes | Yes | Yes | Yes | Yes | Yes |
| 4 | Was the recruitment strategy appropriate to the aims of the research? | Yes | Yes | Yes | Yes | Yes | Yes | Yes | Yes | Yes | Yes | Yes | Yes | Yes |
| 5 | Was the data collected in a way that addressed the research issue? | Yes | Yes | Yes | Yes | Yes | Yes | Yes | Yes | Yes | Yes | Yes | Yes | Yes |
| 6 | Has the relationship between researcher and participants been adequately considered? | No | No | No | Yes | Yes | Yes | Yes | Yes | Yes | Can’t tell | Can’t tell | Can’t tell | Can’t tell |
| 7 | Have ethical issues been taken into consideration? | Yes | Yes | Yes | Yes | Yes | Yes | Yes | Yes | Yes | Yes | Yes | Yes | Yes |
| 8 | Was the data analysis sufficiently rigorous? | Yes | Yes | Yes | Yes | Yes | Yes | Yes | Yes | Yes | Yes | Yes | Yes | Yes |
| 9 | Is there a clear statement of findings? | Yes | Yes | Yes | Yes | Yes | Yes | Yes | Yes | Yes | Yes | Yes | Yes | Yes |
| 10 | How valuable is the research? | ✔ | ✔ | ✔ | ✔ | ✔ | ✔ | ✔ | ✔ | ✔ | ✔ | ✔ | ✔ | ✔ |
| 11 | Overall appraisal | **I** | **I** | **I** | **I** | **I** | **I** | **I** | **I** | **I** | **I** | **I** | **I** | **I** |

**Abbreviations:** **I** – Included
