# Supplementary material for: South Asian immigrants’ and their family carers’ beliefs, practices and experiences of childhood long‐term conditions: An integrative review
Source: J Adv Nurs. 2022 Mar 14;78(7):1897–908. doi: 10.1111/jan.15217 (PMC9314788; doi:10.1111/jan.15217)
Supplement: Supplementary file 4 — Table S4. JBI Critical Appraisal Checklist for analytical cross‐sectional studies. [file JAN-78-1897-s004.docx]

**Supplemental file 4: JBI Critical Appraisal Checklist for analytical cross-sectional studies**

John, A., Bower, K., & McCullough, S. (2016). Indian immigrant parents of children with developmental disabilities: Stressors and support systems. *Early Child Development and Care, 186*(10), 1594-1603. https://doi.org/10.1080/03004430.2015.1116297

| **Sl no** | **Checklist questions** | **Response** |
| --- | --- | --- |
| 1 | Were the criteria for inclusion in the sample clearly defined? | Yes |
| 2 | Were the study subjects and the setting described in detail? | Yes |
| 3 | Was the exposure measured in a valid and reliable way? | NA |
| 4 | Were objective, standard criteria used for measurement of the condition? | Yes |
| 5 | Were confounding factors identified? | No |
| 6 | Were strategies to deal with confounding factors stated? | No |
| 7 | Were the outcomes measured in a valid and reliable way? | Yes |
| 8 | Was appropriate statistical analysis used? | Yes |

Overall appraisal: Included
